# Biomechanical Effects of Socks on Plantar Loading and Foot Function: A Systematic Review

**DOI:** 10.3390/bioengineering13060643

**Published:** 2026-05-29

**Authors:** Roi Painceira-Villar, Paula Cobos-Moreno, Kimas Gudaitis, Alfonso Martínez Nova, Pedro Vicente Munuera-Martínez, Sara García-Oreja

**Affiliations:** 1 Nursing Deparment, Universidad de León, Avda Astorga 15, 24402 Ponferrada, Spain; rpaiv@unileon.es; 2 Nursing Deparment, University Center of Plasencia, University of Extremadura, 10600 Plasencia, Spain; kimas@podoks.com (K.G.); podoalf@unex.es (A.M.N.); 3 Podiatry Department, Faculty of Podiatry, University of Sevilla, 41009 Sevilla, Spain; pmunuera@us.es; 4 Podosauces Podiatry and Health, 03004 Alicante, Spain; saragopodologia@gmail.com; 5 Faculty of Health Sciences, International University of La Rioja, 26006 Logroño, Spain

**Keywords:** socks, biomechanics, plantar pressures, impact peak forces

## Abstract

Background: The biomechanical interaction within the foot–footwear system plays a critical role in load distribution, impact attenuation, and locomotor function. While footwear and orthoses have been widely studied, the role of socks as an interface component remains poorly understood. Objective: This study aimed to systematically review the evidence on the biomechanical effects of socks on human gait, including their influence on plantar pressure, impact attenuation, postural control, and functional performance. Methods: A systematic review was conducted in accordance with PRISMA guidelines and registered in PROSPERO (CRD420261344641). PubMed, Scopus, and Web of Science were searched from inception to March 2026. Experimental, observational, and clinical studies evaluating the effects of socks on foot or lower limb biomechanical variables were included. Study selection, data extraction, and risk of bias assessment were performed independently by two reviewers using adapted Cochrane RoB domains. Results: Twenty-five studies were included. Socks with cushioning or specific designs consistently reduced plantar pressure, particularly in the forefoot, with peak pressure reductions of up to 49% in high-risk populations. Socks also contributed to impact attenuation, reducing peak forces by 6–20%, although this was less than that of footwear. Effects on postural control were heterogeneous: compression socks improved balance and neuromuscular response, whereas thick socks impaired plantar sensitivity and stability. Regarding functional performance, high-friction socks improved performance in sports settings by reducing in-shoe foot displacement, although results were inconsistent. Overall risk of bias was high, mainly due to limitations in randomization. Conclusions: Socks are an active component of the foot–footwear system, capable of influencing key biomechanical parameters, such as plantar pressures. However, further high-quality studies are needed to strengthen the evidence.

## 1. Introduction

Human gait involves a complex biomechanical interaction between the foot, footwear, and the contact surface. During walking and running, the foot is subjected to repetitive loading, generating ground reaction forces that are transmitted throughout the musculoskeletal system [1]. Inadequate distribution of plantar loads and prolonged exposure to elevated pressure levels have been associated with various foot and lower limb pathologies, including metatarsalgia, plantar fasciitis, and overuse injuries [2,3]. These biomechanical alterations are particularly relevant in clinical populations, such as individuals with diabetes mellitus, in whom elevated plantar pressures constitute one of the main risk factors for the development of diabetic foot ulcers [4].

Strategies aimed at reducing plantar pressures and improving load distribution have traditionally focused on footwear design and the use of foot orthoses. Numerous studies have demonstrated that these interventions can increase plantar contact area and reduce peak pressures in regions subjected to high mechanical stress, particularly in the forefoot [5,6]. However, within the foot–footwear system, another component has received relatively limited scientific attention: socks [7].

Socks constitute the direct interface between the foot and footwear; therefore, their structural properties may influence multiple biomechanical variables during locomotion. Factors such as fiber type [8,9], fabric density or material thickness [10,11,12], and cushioning may modify the mechanical interaction between the foot and footwear, affecting plantar pressure distribution, friction, thermoregulation [8,12], impact attenuation, and sensory feedback. One of the most extensively studied biomechanical aspects of the foot–footwear interaction is plantar pressure distribution. Inadequate load distribution, particularly in the forefoot, has been associated with pain, tissue damage, and the development of various foot pathologies. Rather than acting as a passive interface, socks may function as viscoelastic damping elements capable of modulating force transmission and energy dissipation during gait [4,13,14]. In addition to load redistribution, another relevant aspect is impact attenuation during gait; within the energy dissipation chain of locomotion, socks may contribute to attenuating transient mechanical energy before transmission through the musculoskeletal system. During initial heel contact with the ground, transient forces are generated and transmitted through the musculoskeletal system. Although footwear represents the primary determinant of impact attenuation, it has been suggested that intermediate layers may contribute to this process depending on their mechanical properties [15]. Furthermore, socks may influence foot stability within the footwear by modifying friction at the foot–sock–footwear interface, whereas plantar pressure primarily reflects vertical load distribution, and interfacial shear stress refers to tangential frictional forces generated at the foot–sock–footwear interface. Internal foot slippage has been associated with reduced mechanical efficiency and an increased risk of cutaneous injuries, suggesting that friction modulation may have implications for both performance and injury prevention [1].

Despite the growing interest in the biomechanics of the foot–footwear system, the available scientific evidence regarding the specific role of socks remains limited and heterogeneous. Most studies have focused on footwear or foot orthoses, whereas the role of socks as an independent component has been scarcely investigated [16]. Therefore, the aim of the present systematic review was to analyze and synthesize the existing scientific evidence on the biomechanical effects of socks on human gait, including their influence on plantar pressure, impact attenuation, foot stability within footwear, and other relevant biomechanical parameters.

## 2. Materials and Methods

This systematic review was conducted and reported in accordance with the Preferred Reporting Items for Systematic Reviews and Meta-Analyses (PRISMA 2020), Additional details are provided in the Supplementary Materials [17] statement and checklist. The review protocol was prospectively registered in the International Prospective Register of Systematic Reviews (PROSPERO) under the registration number CRD420261344641.

### 2.1. Search Strategy

A systematic search was performed in the electronic databases PubMed, Scopus, and Web of Science from database inception to March 2026. The final search in all databases was conducted on 15 March 2026. The search strategy combined terms related to socks and foot biomechanical variables using Boolean operators (AND, OR). In PubMed, the following strategy was applied: (“Socks”[Mesh] OR sock* OR stocking* OR hosiery) AND (foot OR plantar) AND (“plantar pressure” OR “foot pressure” OR “pressure distribution” OR “peak pressure” OR “pressure–time integral” OR “gait analysis” OR “foot mechanics” OR biomechanic* OR kinematic* OR kinetic*). Equivalent adapted strategies were used in Scopus (TITLE-ABS-KEY) and Web of Science (TS), maintaining the same conceptual structure. No restrictions were applied regarding study design.

### 2.2. Study Selection

Articles meeting the following PICOS-based eligibility criteria were included: (P) healthy individuals or clinical populations; (I) studies evaluating any type of sock or hosiery intervention; (C) barefoot conditions, standard socks, alternative sock designs, or other comparator conditions; (O) biomechanical outcomes related to plantar pressure, impact attenuation, ground reaction forces, postural control, gait, friction, foot stability within footwear, or functional performance; and (S) experimental, observational, laboratory-based, or clinical studies published in peer-reviewed scientific journals in English or Spanish.

Studies were excluded if they did not evaluate biomechanical variables specifically related to sock use, focused exclusively on footwear or foot orthoses without analyzing the independent effect of socks, or were review articles, editorials, conference abstracts, or letters to the editor. Studies that did not meet minimum methodological quality criteria were also excluded.

Title and abstract screening were performed independently by two reviewers. Full-text eligibility assessment was subsequently conducted independently by the same reviewers, and any discrepancies were resolved through discussion with a third reviewer.

### 2.3. Data Extraction and Collection Process

Two reviewers independently assessed full-text articles for eligibility and independently performed data extraction from all included studies. Any disagreements during the selection or extraction process were resolved through discussion and, when necessary, consultation with a third reviewer.

The following data were extracted from each study: (1) author and year of publication; (2) study design; (3) participant characteristics; (4) sample size; (5) type and characteristics of the sock intervention; (6) comparator condition; (7) biomechanical outcomes assessed; (8) biomechanical assessment tools and instrumentation; and (9) main quantitative findings.

Additional variables, including intervention characteristics, experimental conditions, and funding sources when available, were also collected. All biomechanical outcomes compatible with the predefined outcome domains were considered eligible for extraction, including different measures, analyses, and testing conditions reported within each study.

No automation tools or artificial intelligence-assisted screening systems were used during the selection or data extraction processes. When data were unclear or incompletely reported, extraction was limited to the information available in the original publication, and no assumptions beyond the reported data were made.

### 2.4. Methodological Quality Assessment, Risk of Bias and Effect Measures

The methodological quality and risk of bias of the included studies were independently assessed by two reviewers using an adaptation of the Cochrane Risk of Bias (RoB 2) domains. The following aspects were evaluated: randomization process and deviations from intended interventions.

Effect measures were descriptively reported according to the biomechanical variables assessed in each study. These included percentage reductions in plantar pressure, pressure–time integrals, peak force reductions, changes in ground reaction forces, coefficients of friction, center of pressure displacement, electromyographic activity, and other biomechanical performance-related variables. Due to the methodological heterogeneity across studies, pooled quantitative effect estimates were not calculated.

### 2.5. Synthesis Methods

Studies were grouped for synthesis according to the primary biomechanical outcome domain evaluated in each study: plantar pressure, impact attenuation, postural control, and functional performance.

Eligibility for each synthesis category was determined based on the main biomechanical outcomes and experimental objectives reported by the authors. Quantitative findings from individual studies were tabulated to facilitate comparison of intervention effects, biomechanical variables, and methodological approaches.

Due to substantial heterogeneity in study populations, sock designs, biomechanical assessment systems, comparator conditions, and reported outcomes, statistical pooling and meta-analysis were considered inappropriate. Therefore, a qualitative narrative synthesis was conducted.

Where necessary, biomechanical outcomes were descriptively standardized according to the direction of effect (increase, decrease, or no effect) to improve interpretability across studies. Missing summary statistics were not imputed, and only data directly reported in the original studies were included in the synthesis.

## 3. Results

### 3.1. Literature Search

**Figure 1:**
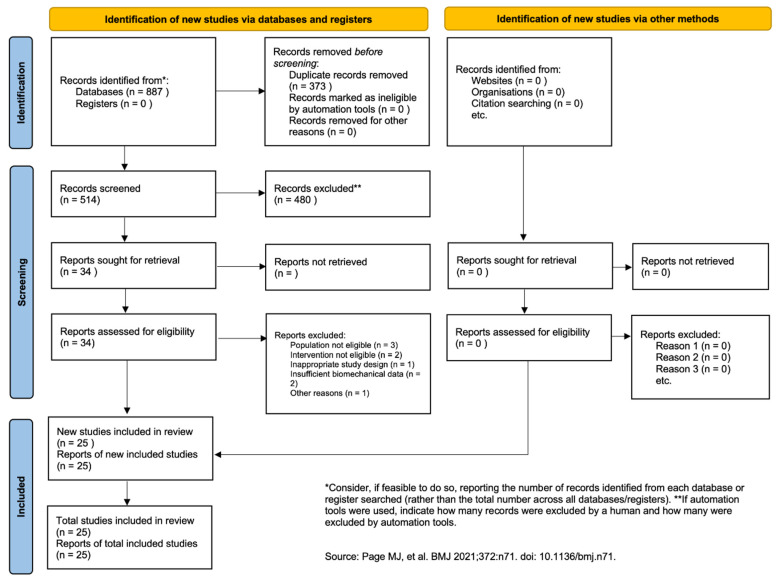
PRISMA 2020 flow diagram of study selection [17].

The results of the study selection process are presented in the PRISMA flow diagram (Figure 1). A total of 887 records were initially identified. After removal of duplicates, 514 unique studies remained. Following title and abstract screening, 480 records were excluded for not meeting the inclusion criteria. A total of 34 full-text articles were assessed for eligibility. During this phase, 9 studies were excluded for failing to meet the predefined inclusion criteria. The main reasons for exclusion included the absence of sock-specific biomechanical outcomes, lack of quantitative biomechanical assessment, insufficient methodological quality, and non-eligible study designs. As a result, a final total of 25 studies were included in the systematic review.

### 3.2. Study Characteristics

The studies were grouped according to the main biomechanical domains evaluated: plantar pressure, impact attenuation, postural control, and functional performance. The characteristics of the included studies, including methodological design, evaluated interventions, and main quantitative biomechanical outcomes, are presented in detail in Table 1.

### 3.3. Methodological Quality and Risk of Bias

**Figure 2:**
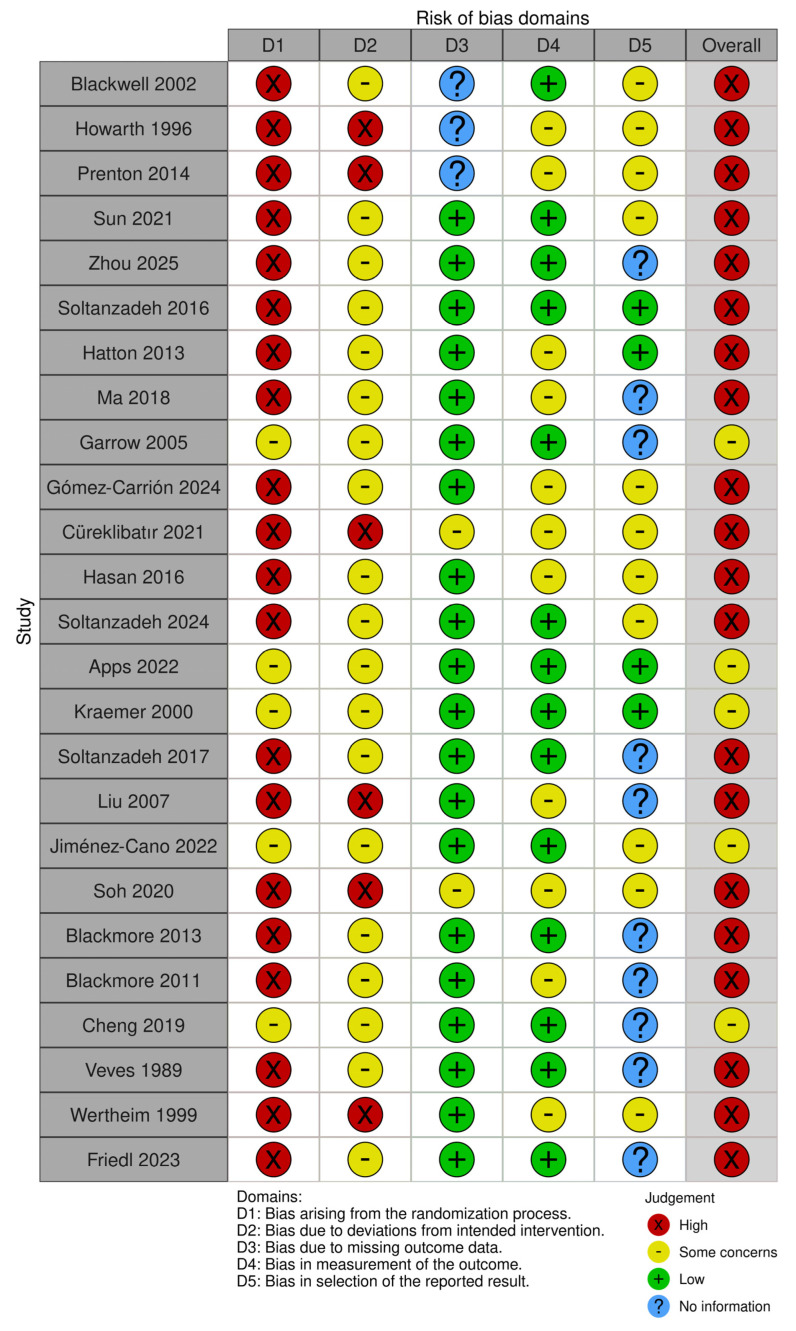
Risk of bias traffic light plot and weighted summary plot [10,11,12,13,14,15,16,18,19,20,21,22,23,24,25,26,27,28,29,30,31,32,33,34,35,36].

**Figure 3:**
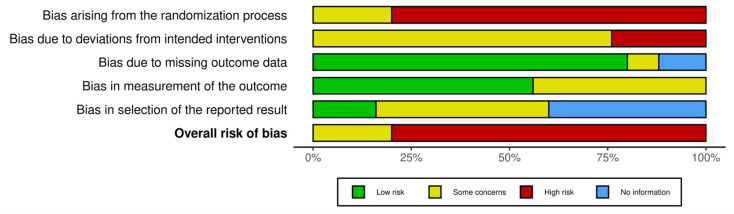
Weighted summary plot of risk of bias.

Figure 2 and Figure 3 present a summary of the risk of bias of the included studies. In the randomization domain, most studies did not report adequate methods for sequence generation or allocation concealment. In several cases, study designs corresponded to laboratory-based experimental studies, non-randomized crossover trials, or observational designs. Consequently, this domain represented the primary determinant of the overall high risk of bias, in accordance with the criteria established by the Cochrane RoB 2 tool. Regarding deviations from intended interventions (such as lack of blinding, participant awareness of sock conditions, or non-standardized intervention delivery), nearly all studies lacked blinding of participants and assessors, which is attributable to the nature of the intervention (use of socks or textile devices). However, given that the outcomes assessed were predominantly objective and biomechanical in nature, the likelihood that these deviations introduced meaningful bias in effect estimation is considered limited.

In the domain of incomplete outcome data, most studies reported complete datasets or minimal attrition, with no evidence that missing data were related to study outcomes, suggesting a low risk of bias in this domain. With respect to outcome measurement, outcomes were assessed using objective instrumentation, including plantar pressure platforms, biomechanical analysis systems, electromyography (EMG), and force sensors. Although in some studies assessors were not blinded, the objective and quantitative nature of these measurements substantially reduces the risk of measurement bias. In the domain of selective reporting, most studies lacked pre-registered protocols or pre-specified statistical analysis plans, limiting the ability to fully exclude the presence of reporting bias.

Overall, although a high global risk of bias was identified—primarily due to limitations in the randomization process—the consistency of findings and the use of objective biomechanical measures support the plausibility of the observed effects. In this context and considering the limited availability of studies in this specific field, the present review is warranted to synthesize the existing evidence, identify knowledge gaps, and guide future research. Nevertheless, the overall certainty of the evidence remains limited and should be taken into account when interpreting the results.

### 3.4. Plantar Pressure

The available evidence consistently demonstrates that socks—particularly those incorporating padded structures or specific therapeutic designs—are capable of reducing plantar pressure, especially in the forefoot. In patients with diabetic neuropathy, padded socks reduced peak plantar pressure by approximately 26% and the pressure–time integral by 29% [18]. Similarly, multilayer socks increased plantar contact area by approximately 14% and reduced forefoot peak pressure by around 10% [19]. In line with these findings, more recent clinical studies have reported reductions of up to 49% in peak plantar pressure in high-risk populations [21].

In healthy populations, different textile configurations have also demonstrated the ability to redistribute plantar load, shifting pressure from high-load regions to areas subjected to lower mechanical stress [10,11,12]. Likewise, the use of socks incorporating three-dimensional offloading elements has been associated with significant reductions in plantar pressure at the central metatarsal heads [26]. However, not all studies reported beneficial effects, with some showing no significant differences between sock types [20], suggesting that efficacy is largely dependent on the structural and functional design of the sock.

### 3.5. Impact Attenuation

With respect to impact attenuation, several studies have demonstrated that socks may contribute—albeit to a lesser extent than footwear—to force absorption during locomotion. Mechanical testing has shown that sports socks can reduce peak impact forces by approximately 6% to 20%, with thickness and degree of cushioning identified as key determinants [23]. Complementary experimental studies have reported that certain textile materials significantly enhance shock absorption compared to barefoot conditions [24]. In runners, specialized socks have also been associated with reductions in maximum plantar pressure and plantar impulse, particularly in the forefoot region [25].

### 3.6. Postural Control

Socks may also influence postural control through alterations in plantar sensory feedback mechanisms. Compression socks have been shown to improve balance recovery following perturbations and to reduce muscle activity, suggesting an optimization of afferent sensory input [27]. In contrast, thick socks have been associated with reduced plantar sensitivity and increased center of pressure displacement, indicating impaired postural control [34]. In older adults, standard socks were associated with more cautious gait patterns, whereas non-slip socks demonstrated behavior more closely resembling barefoot walking [33].

### 3.7. Functional Performance

In the sports domain, modulation of friction at the foot–sock interface appears to play a relevant role. High-friction socks increased the coefficient of friction and improved performance in agility tasks, reducing in-shoe foot displacement [32]. Similarly, socks incorporating compression and surface texturing improved performance-related variables, such as ball velocity in soccer [30]. However, findings were not consistent across studies, suggesting that the effects depend on task specificity, athlete level, and interaction with footwear [31].

### 3.8. Other Biomechanical Effects

Finally, some studies evaluated specific biomechanical effects. A biomechanical sock significantly reduced the force required to activate the foot’s windlass mechanism (13.33 ± 3.54 N; *p* < 0.001), suggesting a potential improvement in the functional efficiency of the plantar arch [36]. Conversely, socks designed to assist dorsiflexion in patients with foot drop did not demonstrate objective improvements in gait parameters, although they were positively perceived from a subjective standpoint [35].

Each study was classified according to the primary biomechanical domain assessed. The distribution of studies and synthesis of observed effects is presented in Table 2.

## 4. Discussion

The present systematic review analyzed the available evidence regarding the biomechanical effects of socks during human locomotion, identifying that their structural design can meaningfully influence variables such as plantar pressure, impact attenuation, postural control, and functional performance. Overall, the findings suggest that socks should not be considered a passive element within the foot–footwear system, but rather an active component capable of modifying the mechanical interaction between the foot and the environment.

One of the most consistent findings was the reduction in plantar pressure associated with the use of padded or specifically engineered socks. This effect appears particularly relevant in clinical populations, such as individuals with diabetes, where a reduction in forefoot peak pressures is a key factor in ulcer prevention. These findings are consistent with previous studies demonstrating the relationship between elevated plantar pressures and the development of foot lesions, particularly in the context of peripheral neuropathy [4,29]. From a biomechanical perspective, the redistribution of plantar loads observed with different textile designs may be explained by changes in contact surface area and material cushioning properties, thereby reducing stress concentrations in specific regions of the foot.

Regarding impact attenuation, the results indicate that socks may contribute complementarily to footwear in absorbing forces during walking and running. Although their effect is smaller compared to that of the shoe sole, the evidence suggests that thicker or cushioned materials can reduce both peak impact and loading rate. This finding aligns with biomechanical studies linking reduced impact forces to a lower risk of overuse injuries in the lower limb [1,16].

In contrast, findings related to postural control and sensory feedback were more heterogeneous. While compression socks appear to enhance stability and neuromuscular response, thick socks may reduce plantar sensitivity and compromise balance. These results can be interpreted in light of the fundamental role of plantar sensory input in postural control and gait regulation [22,28]. Modulation of friction and compression at the foot–sock interface may alter sensory perception and, consequently, the motor strategies adopted during locomotion.

In the sports context, socks with high-friction properties demonstrated positive effects on performance, particularly in tasks requiring rapid changes in direction. This effect may be explained by improved force transmission between the foot and footwear, reducing internal slippage and optimizing mechanical efficiency. However, variability across studies suggests that these effects are influenced by multiple factors, including activity type, athlete level, and footwear characteristics, thereby limiting generalizability. Potential sources of heterogeneity include differences in sock material composition, compression levels, cushioning thickness, participant characteristics, footwear conditions, gait tasks, and biomechanical assessment systems. These methodological differences likely contributed to the variability observed across studies.

Despite these promising findings, several limitations of this review should be acknowledged. First, there is considerable heterogeneity among the included studies in terms of experimental design, population characteristics, sock type, and outcome measures, which limits direct comparability. Second, many studies included small sample sizes, reducing statistical power and generalizability. Furthermore, most studies were cross-sectional or laboratory-based experimental designs, with limited availability of longitudinal studies or high-quality randomized clinical trials.

Future research should therefore focus on the development of well-designed randomized controlled trials with larger sample sizes, as well as on the standardization of biomechanical outcome measures. It would also be relevant to investigate the long-term effects of different types of socks in both clinical and athletic populations, as well as their interaction with various types of footwear.

These methodological differences should be considered when interpreting the consistency, comparability, and generalizability of the findings across studies.

## 5. Conclusions

Socks should be considered an active component within the foot–footwear system, with the capacity to modify plantar pressure distribution, impact attenuation, and functional performance. Designs incorporating cushioning or specific structural elements appear to reduce peak plantar pressures, with potential clinical implications for injury prevention. However, the available evidence remains heterogeneous, particularly regarding postural control and performance outcomes. This highlights the need for higher methodological quality studies.

## Figures and Tables

**Table 1:** Characteristics included studies and quantitative biomechanical outcomes of sock interventions.

Study	Sample	Study Design	Intervention	Comparator	Outcome	Key Quantitative Results
**Veves et al. (1989)** [18]	*n* = 27	Experimental crossover study with pedobarography	Padded hosiery	Barefoot/usual hosiery	Plantar pressure	↓ 26% peak; ↓ 29% PTI
**Garrow et al. (2005)** [19]	*n* = 19	Repeated-measures in-shoe pressure study	Multilayer socks	Standard socks	Plantar pressure	↓ 9% total; ↓ 10.2% forefoot; ↑ 14.2% contact
**Blackwell et al. (2002)** [20]	*n* = 21	Comparative in-shoe pressure study	Diabetic sock	Dress sock/control	Plantar pressure	No significant differences
**Cüreklibatır et al. (2021)** [14]	*n* = 11	Prospective longitudinal clinical study	Diabetic sock	Follow-up	Plantar pressure	↓ M4–M5; no global effect
**Soh et al. (2020)** [21]	*n* = 31	Prospective non-randomized clinical trial	StepEase socks	Barefoot	Plantar pressure	↓ 22–49%
**Blackmore et al. (2011)** [22]	*n* = 5	Repeated-measures laboratory study	Running sock	Control	GRF	↓ 0.075 BW
**Blackmore et al. (2013)** [23]	7 conditions	Mechanical impact testing study	Athletic socks	No sock	Impact	↓ 6–20%
**Howarth & Rome (1996)** [24]	*n* = 1	Single-subject repeated-measures study	Cushioned socks	Barefoot	Impact attenuation	↑ (*p* < 0.05)
**Zhou et al. (2025)** [25]	*n* = 10	Experimental repeated-measures study	Running socks	Standard	Pressure/impact attenuation	↓ (*p* < 0.05)
**Soltanzadeh et al. (2016)** [12]	*n* = 10	Experimental gait analysis study	Sock structures	Barefoot	Dynamic pressure	↓ M1 (*p* < 0.05)
**Soltanzadeh et al. (2017)** [11]	*n* = 10	Experimental standing study	Cotton structures	Barefoot	Static pressure	↓ M1–M2
**Soltanzadeh et al. (2024)** [10]	*n* = 10	Experimental + modelling study	Stitch length socks	Barefoot	Pressure	↓ forefoot
**Jiménez-Cano et al. (2022)** [26]	*n* = 38	Randomized repeated-measures study	3D sock	Control	Pressure	↓ M2–M3
**Cheng & Xiong (2019)** [16]	*n* = 16	Controlled laboratory crossover study	Compression socks	No compression	Biomechanics	↑ ankle moment
**Sun et al. (2021)** [27]	*n* = 12	Repeated-measures experimental study	Compression socks	Control	Balance/EMG	↓ EMG
**Kraemer et al. (2000)** [15]	*n* = 12	Experimental controlled study	Compression hosiery	Control garments	Physiology	↓ edema
**Wertheim et al. (1999)** [28]	*n* = 6	Experimental laboratory study	Compression stockings	Movement conditions	Pressure	Varies
**Liu et al. (2007)** [29]	*n* = 6	Experimental laboratory study	Compression stockings	Postures	Pressure	Significant changes
**Hasan et al. (2016)** [30]	*n* = 12	Experimental controlled study	Compression + texture	Control	Performance	↑ ball velocity
**Friedl et al. (2023)** [31]	*n* = 12	Randomized crossover study	Grip socks	Compression socks	Friction	↑ COF +9.3%; no ↓ sliding
**Apps et al. (2022)** [32]	*n* = 20	Repeated-measures experimental study	Grip socks	Regular socks	Performance	COF 1.17 vs. 0.60
**Hatton et al. (2013)** [33]	*n* = 15	Repeated-measures experimental study	Nonslip socks	Standard socks	Gait	Standard socks ↓ speed
**Ma et al. (2018)** [34]	*n* = 14	Repeated-measures experimental study	Thick socks	Barefoot	Balance	↑ COP; ↓ sensation
**Prenton et al. (2014)** [35]	*n* = 2	Single-case experimental design (A–B)	Dorsiflex sock	Control	Gait	No improvement
**Gómez-Carrión et al. (2024)** [36]	*n* = 30	Repeated-measures laboratory study	Biomechanical sock	Control	Jack test	13.33 ± 3.54 N (*p* < 0.001)

Abbreviations: PTI, pressure–time integral; GRF, ground reaction force; BW, body weight; COP, center of pressure; COF, coefficient of friction; M1–M5, metatarsal heads 1–5; ↓ decrease; ↑ increase.

**Table 2:** Summary of biomechanical effects of sock interventions.

Biomechanical Domain	Number of Studies (n)	Direction of Effect	Magnitude of Effect	Consistency
Plantar pressure	12	Decrease	9–49% reduction.	High
Impact attenuation	4	Decrease	6–20% reduction.	Moderate
Postural control	3	Mixed	Variable.	Low
Functional performance	4	Increase/Mixed	Improved friction-related performance outcomes; variable effects across studies.	Low–Moderate
Other biomechanical effects	2	Mixed	Task-specific.	Low

## Data Availability

The data supporting reported results can be found at https://www.crd.york.ac.uk/PROSPERO/view/CRD420261344641 (accessed on 11 May 2026).

## Supplementary Materials

The following supporting information can be downloaded at https://www.mdpi.com/article/10.3390/bioengineering13060643/s1. PRISMA 2020 Checklist.

## Abbreviations

The following abbreviations are used in this manuscript:

PRISMA	Preferred Reporting Items for Systematic Reviews and Meta-Analyses
EMG	Electromyography
